# Innovations in neurosurgical education: the role of neurosurgical labs at neuroscience hospital of Baghdad in enhancing surgical skills

**DOI:** 10.1186/s12909-025-07535-7

**Published:** 2025-07-06

**Authors:** Moneer K. Faraj, Wamedh E. Matti, Rania H. Al-Taie

**Affiliations:** 1 Surgery Department, College of Medicine, University of Baghdad, Baghdad, Iraq; 2 Department of Neurosurgery, Neuroscience Hospital, Baghdad, Iraq; 3 Department of Surgery, College of Medicine, University of Mustansiriyah, Baghdad, Iraq

**Keywords:** Education, Innovation, Neurosurgical lab, Simulation-based training, Skill development

## Abstract

**Background:**

The field of neurosurgical education has seen significant advancements in recent years. However, the distribution of advanced neurosurgical education remains uneven, particularly in developing countries like Iraq. This study aims to evaluate the effectiveness of the neurosurgical lab at Neuroscience Hospital in Baghdad, focusing on its impact on surgical precision, skill and acquisition among neurosurgery residents.

**Method:**

A cross-sectional survey targeted neurosurgeons, neurosurgical residents, and medical students actively utilizing the lab. The survey assessed the frequency of lab use, the impact of lab resources on clinical practice, and skill development. Data was analyzed using descriptive statistics and chi-square tests to explore associations between variables such as lab usage frequency, satisfaction, and professional development.

**Results:**

Forty participants responded to the questionnaire. The results revealed that 60% of the participants expressed high satisfaction. The microscope and training models were considered highly impactful, with 80% of participants rating these resources as 4 or 5 on a 5-point scale. The chi-square tests revealed a statistically significant relationship between lab use frequency and satisfaction (χ² = 11.4, *p* = 0.010).

**Conclusion:**

The neurosurgical lab at Neuroscience Hospital has proven to be an essential resource for enhancing surgical skills and promoting innovation. The findings highlight the need for continued integration of advanced technologies like 3D printing to improve neurosurgical training and outcomes in resource-limited settings.

**Supplementary Information:**

The online version contains supplementary material available at 10.1186/s12909-025-07535-7.

## Introduction

Neurosurgical education has been developing rapidly. This includes an upward rise in training methodologies and state-of-the-art technologies in most neurosurgical centers worldwide. On the one hand, while there are approximately 49,940 practicing neurosurgeons worldwide, their distribution remains uneven, with developing countries finding access to state-of-the-art neurosurgical education and training facilities to be the biggest challenge [1].

As in the case of the Neuroscience Hospital in Baghdad, the neurosurgical laboratory becomes crucial to bridge these gaps and provides residents with simulation-based exposure that can enhance technical skills while improving patients’ reports of outcomes. Simulation technology in neurosurgical education has significantly improved both didactic knowledge and practical competence [2]. This represents a shift toward more structured and competency-based training, unlike previous apprenticeship models, whereby the trainee can practice high-risk procedures in a controlled environment [3]. Indeed, creating entities such as the Barrow Innovation Center has shown that innovation programs driven by residents but supported by faculty are critical in allowing neurosurgeons to take an active role in medical device development, procedural advancement, and new treatment modalities [3]. These labs enable technical skills and inculcate a culture for continuous improvement and critical problem-solving, an element necessary for future neurosurgeons. Despite war and adversity, Iraq’s neurosurgical field has advanced significantly, overcoming resource limitations and geopolitical instability. Innovations such as deep brain stimulation and Gamma Knife radiosurgery have been introduced, demonstrating that progress is possible despite challenges. Additionally, neurosurgical education has been transformed through specialized training programs and the integration of advanced technologies like 3D printing to equip future neurosurgeons. These efforts highlight a commitment to innovation, resilience, and the continuous advancement of neurosurgical practice in Iraq [4].

This study aims to evaluate the effectiveness of the neurosurgical simulation lab at Neuroscience Hospital in Baghdad in enhancing microsurgical skills, anatomical precision, innovation capacity, and professional development among neurosurgery residents, medical students, and neurosurgeons. Rather than assessing direct clinical outcomes, the study focuses on participants’ perceptions and satisfaction, exploring how frequent engagement with simulation tools correlates with improvements in educational and research competencies. By providing both quantitative and qualitative insights, this study contributes novel evidence on the role of simulation-based neurosurgical training in resource-limited environments.

## Method

It was designed to assess the effectiveness of the neurosurgical lab in Neuroscience Hospital, Baghdad, given acquisition, innovation of skills, and professional development among its users. A cross-sectional survey has been distributed to professionals attached to this lab.

### Study design

This study employed a quantitative cross-sectional survey design to seek detailed responses from neurosurgeons, neurosurgical residents, and medical students. The survey lasted for two months to consider the lab resources and their impacts on various professional competencies to be elicited, including surgical skills and innovative problem-solving, among other competencies.

The training schedule in the neurosurgical lab is a prime example of structured and mentor-driven skill development. A series of critical weekly sessions offers practical experience ranging from basic microsurgical techniques to complex anatomical challenges using high-fidelity simulation tools. Neurosurgical lab sessions were led by recognized neurosurgeons and senior residents. The trainers were available on a weekly basis to ensure structured guidance during the hands-on practice sessions of participants. They allowed participants to learn the skill through detailed demonstrations, supervised skill acquisition, and personalized feedback; instead, participants followed a mentor-led approach rather than relying on peer-to-peer teaching.

### Participants

Respondents were chosen based on their active involvement in neurosurgical procedures and the continuous use of resources in the lab. The 40 respondents came from professionals such as neurosurgeons, neurosurgical residents, and medical students. Also, participants were grouped based on their years in neurosurgery, ranging from zero to over 15 years.

### Survey structure and data collection

This was a survey of a set of Likert-scale questions and categorical variables. The survey is divided into key sections to assess various aspects of lab usage and its impact on professional development.

The survey used in this study was specifically developed by the authors for the purpose of this research and has not been previously published or validated elsewhere (Supplementary File 1).

They were asked to identify how often they used the facilities in the lab, which ranged from ‘Occasional’ to ‘Weekly,’ reflecting the use patterns within the lab. In addition, respondents rated the usage impact of specific resources in the lab, such as a microscope, training models, 3D printers, medical books, and non-medical books. The impact question was based on a 5-point Likert scale, ranging from one, indicating minimal impact, to five, indicating the aspect mentioned had a highly significant impact on their clinical practice. Skill Development and Professional Growth section evaluated participants’ perception of the lab’s contribution to their development in important virtues: microsurgical skills, precision in anatomy, awareness of three-dimensional space, and problem-solving with the help of 3D printing. Additional questions focused on diagnostic accuracy using the microscope and training models. Moreover, to gauge the broader professional impact of the lab, respondents rated how well the lab supported their research capabilities and leadership development, including decision-making fostered by non-medical resources available in the lab, such as books on leadership, innovation, cognitive skills, and teamwork. The survey was distributed electronically to all eligible participants, with responses collected anonymously to ensure unbiased feedback.

### Statistical analysis

Data were analyzed using the Jamovi statistical software (Version 2.3) ​ [5]. Descriptive statistics, medians, standard deviations, and frequency distributions were generated for all the items on the survey. These described the general trends and dispersion in responses given by participants.

The association between categorical variables was explored using contingency tables. For example, χ² tests compare the frequency of lab use to overall satisfaction with the lab experience. Years of neurosurgical experience vs. perceived impact of lab resources used χ² tests. Statistical significance was set at a *p* < 0.05 level.

### Ethical considerations

This study involved a voluntary, anonymous survey of medical professionals and did not include patient data or clinical interventions. As such, formal ethical approval was not required under ethical regulations in Baghdad University-College of Medicine. All participants provided informed consent before commencing the survey. All participants provided informed consent before commencing the survey. The study was conducted in accordance with the ethical standards of the institutional and national research committees and with the 1964 Helsinki Declaration and its later amendments or comparable ethical standards. Strict anonymity and confidentiality were maintained throughout the research process, with data securely stored.

## Results

This study surveyed 40 participants who actively use the neurosurgical lab at Neuroscience Hospital in Baghdad. The participants included neurosurgeons, neurosurgical residents, and medical students, all of whom provided feedback on the lab’s impact on their clinical practice, skill development, and research capabilities (Table 1).

**Table 1:** Demographic characteristics of participants in neurosurgical lab usage study

Demographic Variable	Category	*N*	Percentage (%)
Age	Under 25 years	4	10.0
	25–30 years	8	20.0
	31–35 years	16	40.0
	36–40 years	4	10.0
	Over 50 years	8	20.0
Gender	Male	32	80.0
	Female	8	20.0
Current Position	Neurosurgeon	12	30.0
	Neurosurgical Resident	24	60.0
	Medical Student	4	10.0
Years of Experience in Neurosurgery	0–2 years	8	20.0
	3–5 years	20	50.0
	11–15 years	4	10.0
	More than 15 years	8	20.0
Frequency of Lab Use	Weekly	28	70.0
	Daily	4	10.0
	Monthly	4	10.0
	Occasionally	4	10.0

### Descriptive statistics

The sample comprised 80% males (*n* = 32) and 20% females (*n* = 8). Participants’ years of experience in neurosurgery varied, with 50% of respondents (*n* = 20) having 3–5 years of experience, while 20% (*n* = 8) had less than two years of experience. Furthermore, 20% (*n* = 8) had more than 15 years of experience, and 10% (*n* = 4) had 11–15 years of experience.

Most participants (70%, *n* = 28) reported using the lab every week, while 10% (*n* = 4) reported daily use, 10% (*n* = 4) monthly use, and another 10% (*n* = 4) occasional use.

### Impact of lab resources on clinical practice

Participants rated the impact of specific lab resources on their practice using a Likert scale. The microscope was highly rated, with 40% of respondents (*n* = 16) rating its impact as 5 (maximum impact) and another 40% (*n* = 16) rating it as 4. Similarly, training models were considered highly impactful, with 30% (*n* = 12) of participants rating their impact as 5 and 40% (*n* = 16) rating them as 4.

Regarding the 3D printers, 20% (*n* = 8) rated their impact as 5, while 40% (*n* = 16) rated them as 4. However, 30% (*n* = 12) rated 2, indicating this resource’s lesser perceived impact.

Medical books were also viewed as important resources, with 40% (*n* = 16) of participants rating their impact as 5 and 50% (*n* = 20) rating them as 4. In contrast, non-medical books had more varied ratings, with 40% (*n* = 16) providing a score of 3 and another 40% (*n* = 16) providing a maximum rating of 5 (Table 2).

**Table 2:** Impact of microscope, training models, 3D printers, and books in practice

Tool/Resource	Rating 3 (Moderate Impact)	Rating 4 (High Impact)	Rating 5 (Very High Impact)	*N*
Microscope	8 (20%)	16 (40%)	16 (40%)	40
Training Models	12 (30%)	16 (40%)	12 (30%)	40
3D Printers	4 (10%)	16 (40%)	8 (20%)	40
Medical Books	4 (10%)	20 (50%)	16 (40%)	40
Non-Medical Books	4 (10%)	16 (40%)	16 (40%)	40

### Skill development

The lab was perceived as highly effective in enhancing participants’ skills. Regarding microsurgical skills, 30% (*n* = 12) rated the lab’s contribution as 5, while 50% (*n* = 20) rated it as 4. For anatomical precision and spatial awareness, 70% (*n* = 28) of participants rated the lab’s impact as 4, and 20% (*n* = 8) rated it as 5.

When assessing innovative problem-solving skills through 3D printing, 40% (*n* = 16) rated its impact as 4, while 30% (*n* = 12) rated 2, reflecting some variability in perceptions of this specific resource.

Diagnostic accuracy using the microscope and training models was rated positively, with 50% (*n* = 20) rating its impact as 4 and 30% (*n* = 12) providing a rating of 5 (Table 3).

**Table 3:** Microsurgical skills, anatomical precision, and Problem-Solving skills

Skill/Competency	Rating 3	Rating 4	Rating 5	*N*
Microsurgical Skills	8 (20%)	20 (50%)	12 (30%)	40
Anatomical Precision and Spatial Awareness	4 (10%)	28 (70%)	8 (20%)	40
Innovative Problem-Solving Skills (3D Print)	8 (20%)	16 (40%)	4 (10%)	40
Diagnostic Accuracy (Microscope & Models)	8 (20%)	20 (50%)	12 (30%)	40

### Research and leadership skills

Regarding research skills supported by the lab’s resources, 30% (*n* = 12) rated the lab’s contribution as 5, while 40% (*n* = 16) rated it as 4. Leadership and decision-making skills fostered by the lab were similarly rated, with 50% (*n* = 20) of participants scoring 4 and 20% (*n* = 8) rating the impact as 5.

### Overall satisfaction

**Fig. 1:**
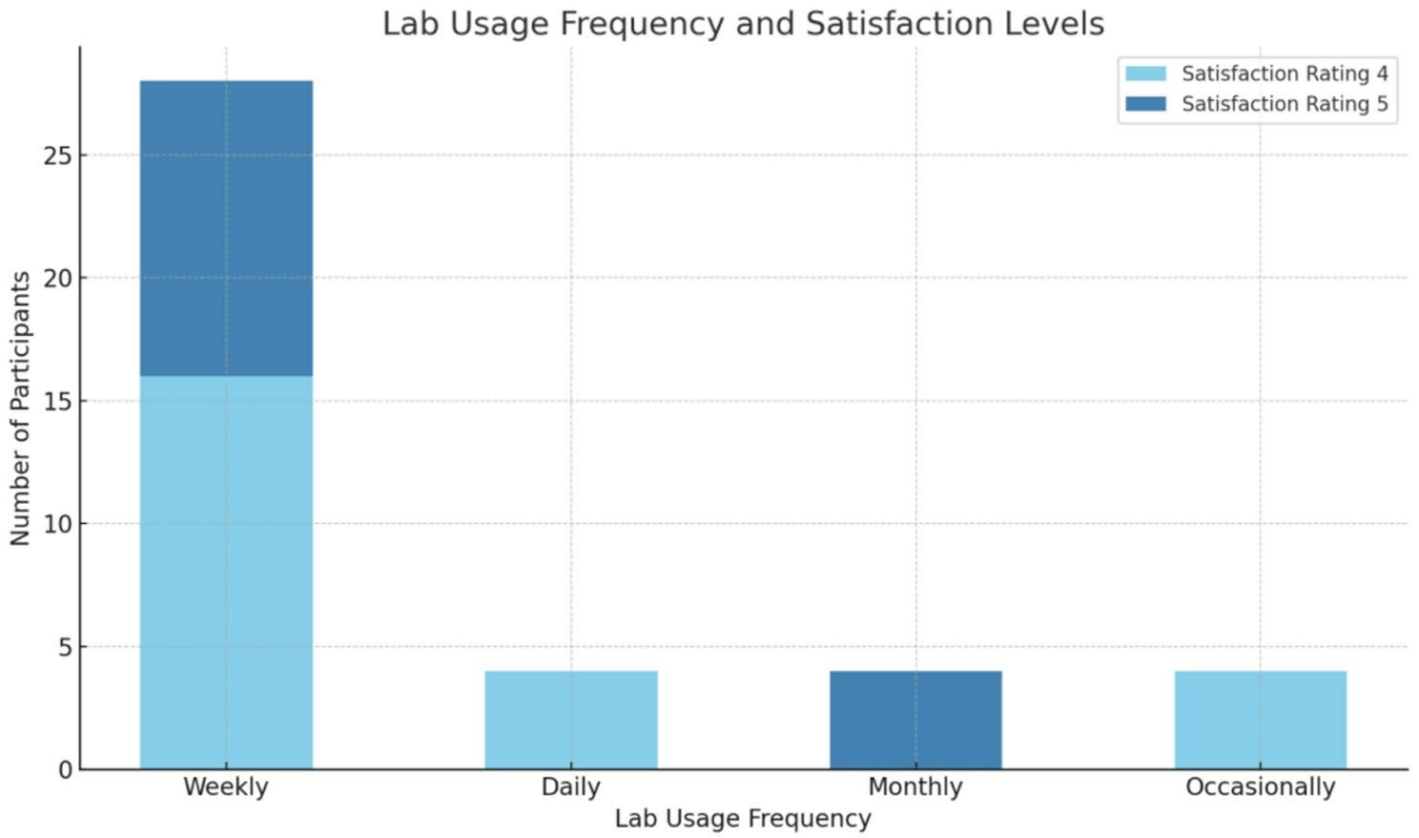
Relationship Between Lab Usage Frequency and Satisfaction Levels in Neurosurgical Practice

**Fig. 2:**
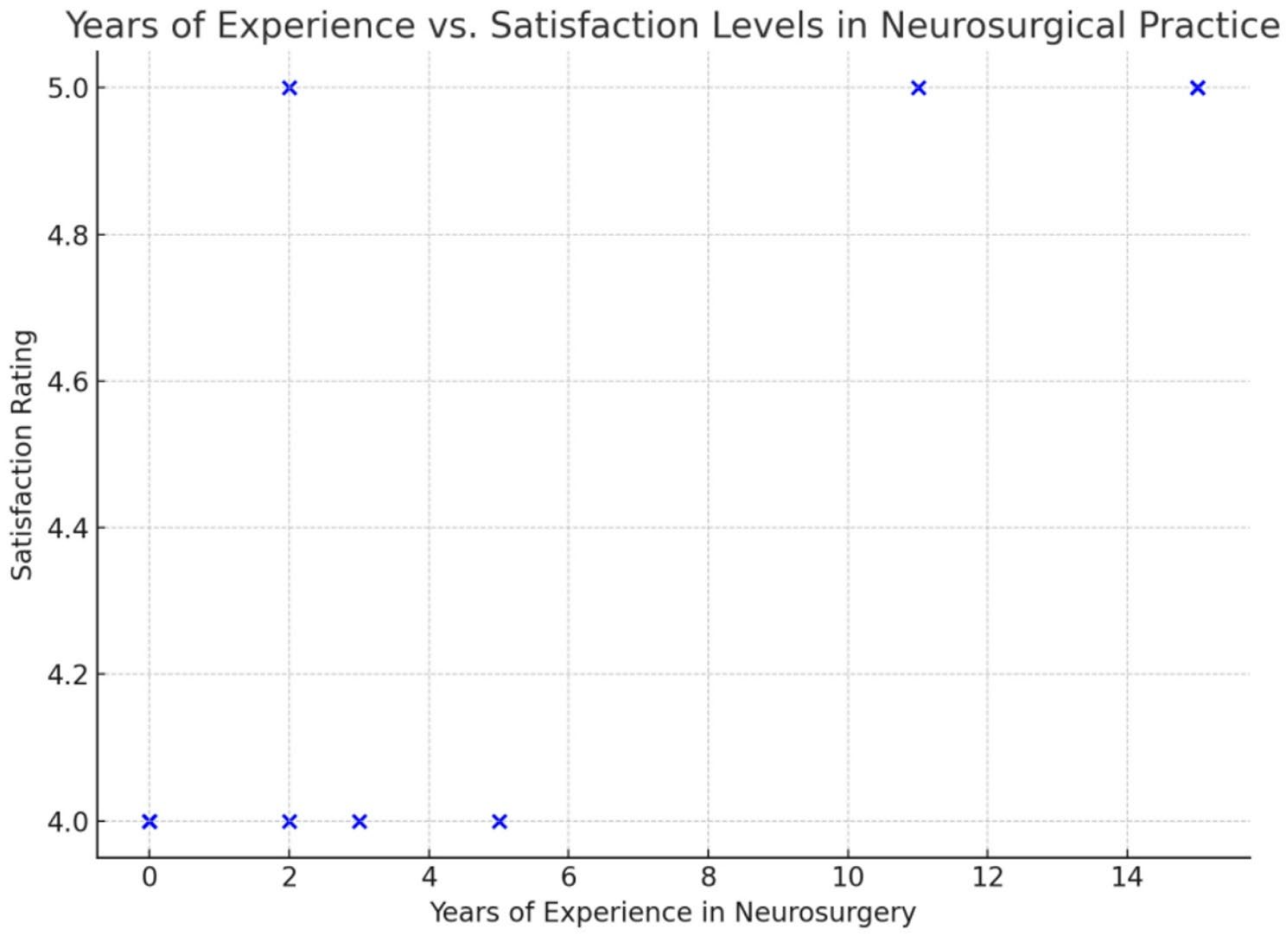
Correlation Between Years of Experience and Satisfaction Levels in Neurosurgical Practice

When asked about their overall satisfaction with the lab experience, 40% of participants (*n* = 16) rated their satisfaction as 5, while 60% (*n* = 24) rated it as 4. A chi-square test revealed significant associations between satisfaction and several variables, including the frequency of lab use (χ² = 11.4, *p* = 0.010) and years of experience in neurosurgery (χ² = 11.7, *p* = 0.009). Additionally, the lab’s role in encouraging innovation was rated as highly effective, with 40% (*n* = 16) of respondents rating it as 5 and 60% (*n* = 24) rating it as 4. A significant relationship was found between the frequency of lab usage and the perception of the lab’s role in fostering innovation (χ² = 11.4, *p* = 0.010) (Figs. 1 and 2).

## Discussion

The history of neurosurgery in Iraq is deeply rooted in ancient Mesopotamia, where early forms of surgical intervention, such as abscess drainage, were documented on cuneiform tablets​. The scientific structure of neurosurgery in Iraq took shape in the 1950s, with the first elective neurosurgical procedure being carried out by Dr. Najeeb Al-Yaaqubi and was further formalized in 1966 by Dr. Saad Al-Witry, considered the father of Iraqi neurosurgery. A milestone was passed when 1972 the Neurosurgery Teaching Hospital was inaugurated in Baghdad; this became a central focal point for neurosurgical training and practice [6]. The history of neurosurgery development took another course in the Kurdistan region of Iraq when international collaborations aimed at building local neurosurgical capacities in cities like Duhok started in 2012 [7].

**Fig. 3:**
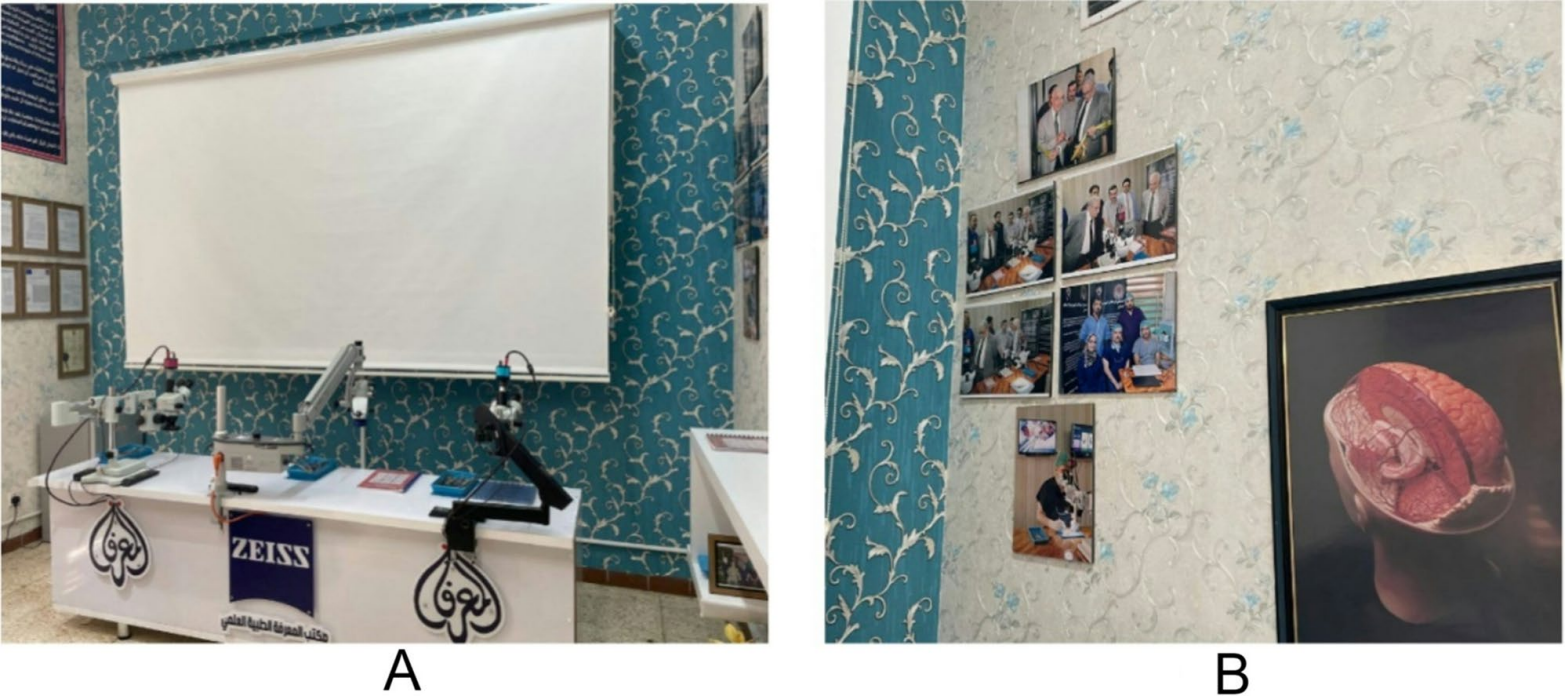
Neurosurgical Training Facilities at Neuroscience Hospital, Baghdad. (**A**) Microscope stations and 3D training models used by residents for hands-on neurosurgical simulation and skill development. (**B**) A collection of photos showcasing collaborative workshops and innovation sessions between neurosurgery residents, faculty members, and international visitors, underscoring the importance of mentorship and skill sharing in the lab

**Fig. 4:**
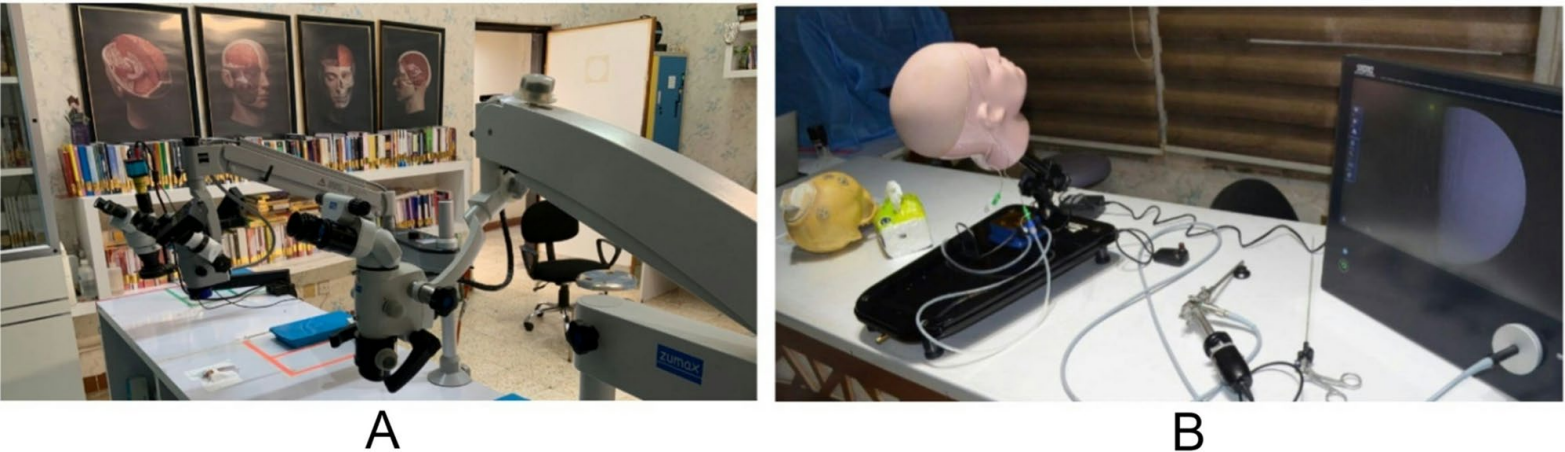
Advanced Neurosurgical Training Equipment at Neuroscience Hospital, Baghdad. (**A**) High-resolution microscopes used for neurosurgical simulations, enabling residents to practice precision techniques in a controlled environment. Anatomical posters and reference books support theoretical learning alongside practical skills. (**B**) Endoscopic training setup featuring a head model and monitor, simulating real-time surgical procedures for enhancing skills in minimally invasive neurosurgery

**Fig. 5:**
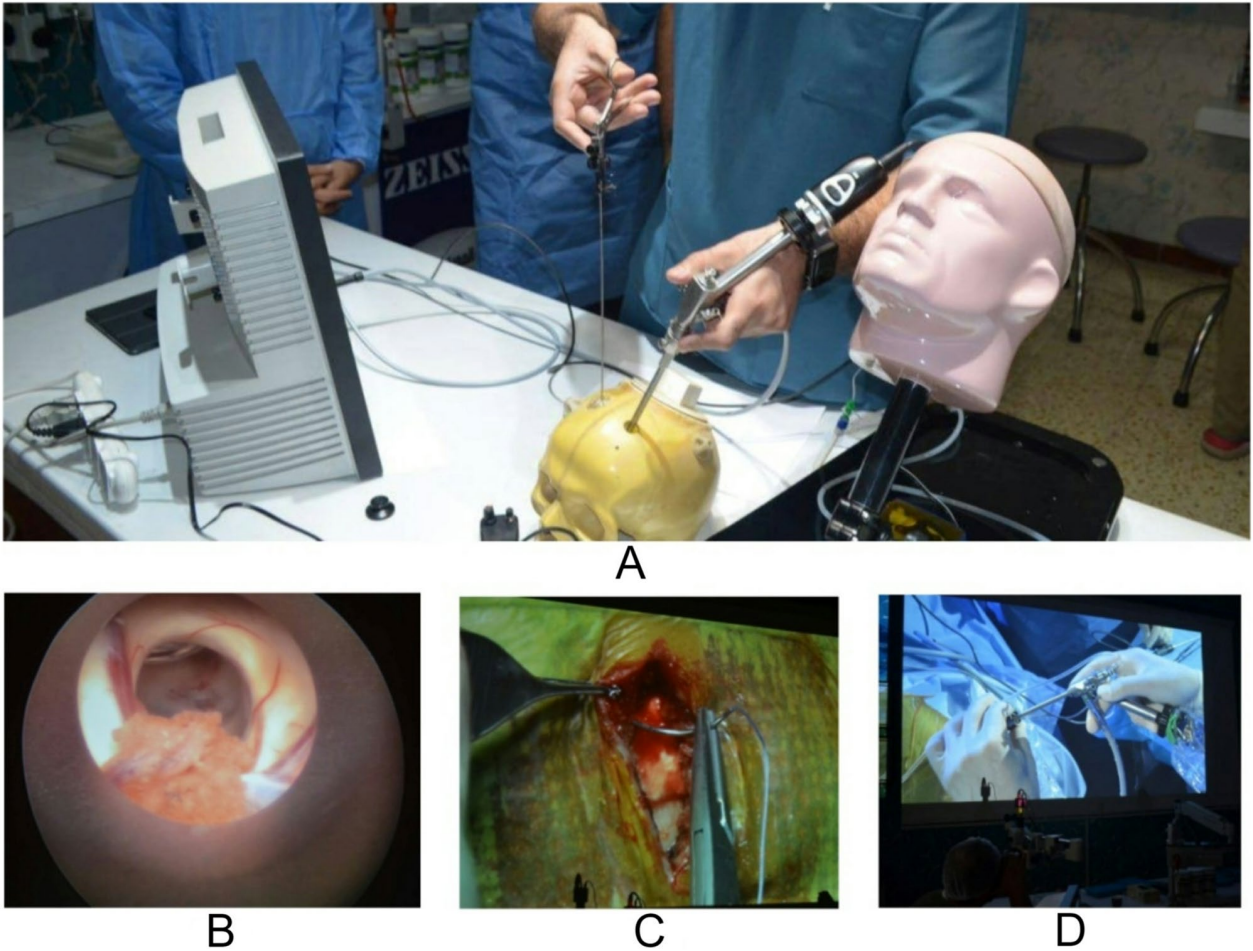
Endoscopic and Microsurgical Simulation in Neurosurgical Training at Neuroscience Hospital, Baghdad. (**A**) Hands-on endoscopic training using a skull model, allowing residents to practice minimally invasive neurosurgical techniques. (**B**) Endoscopic view displaying the needed anatomy during a simulated procedure, enhancing visual-spatial understanding. (**C**) Microsurgical practice with high magnification, providing precision skill training in surgical anatomy and dissection. (**D**) Live demonstration of microsurgical techniques projected on a screen, used for educational purposes in ongoing resident workshops and seminars

The research findings are useful in understanding the role of the neurosurgical virtual laboratory at Neuroscience Hospital, Baghdad (Figs. 3, 4 and 5). The neuroscience hospital at Baghdad uses a stepwise, competency-based educational model in the neurosurgical lab. This was mainly developed through simulation-based training on clinically relevant scenarios. Iterative practice further reinforces learning, whereby immediate feedback from mentors during the process provides participants with opportunities to refine techniques and decision-making. It provided a very excellent avenue of professional growth between mentors and mentees. The mentors then took the mentees through iterative learning cycles of providing personalized feedback and clinical insights that could help fine-tune surgical precision and decision-making. Teaching in the neurosurgical lab was led by experienced neurosurgeons and senior residents with specialized expertise in simulation-based training.

A considerable proportion of participants (70%) reported weekly lab use, indicating that the facility is integral to their training and practice. Moreover, overall satisfaction with the lab experience was high, with 60% rating their satisfaction as 4 and 40% rating it as 5. This suggests that the lab is meeting the expectations of its users across various levels of experience. Notably, the chi-square test revealed a statistically significant association between satisfaction and the frequency of lab use (*p* = 0.010), highlighting that those who use the lab more frequently are more likely to be satisfied with their experience. Interestingly, satisfaction was not significantly influenced by gender (*p* = 0.519), indicating that both male and female participants had comparable satisfaction levels. However, years of neurosurgical experience significantly affected satisfaction (*p* = 0.009), with participants having more than 15 years of experience showing the highest levels of satisfaction. The gender distribution was skewed toward male participants, reflecting the broader gender imbalance currently observed in the neurosurgical field in our region.

The results demonstrate the high perceived educational value of various lab resources. The microscope and training models were consistently rated highly, with 40% of participants giving a maximum score of 5 for both tools. The impact of the 3D printers is rated lower: 20% gave a score of 5, and 40% gave a score of 4. This suggests variability in either utilization or perception of this resource. For medical textbooks, the rating was incredibly positive for 40% of the respondents.

In the article about the surgical skills in neurosurgical residency training, Liu et al. [8] focused on the especially important role of neurosurgical residency training in the context of surgical skills laboratories. They sought to enhance residents’ technical skills in complex skull base operations through a cadaveric-based, structured dissection curriculum and modern equipment at Cleveland Clinic. Furthermore, three-dimensional printing has evolved to be a robust neurosurgical education and anatomy training tool. Thiong’o et al. (2021) [9] describe the role that 3D printing plays in neurosurgical simulation, including skull base surgery and vascular procedures, to practice complex surgical skills outside the operating theater. It has proved beneficial in decreasing the learning curve for difficult procedures. Also, Baskaran et al. (2016) [10] point out that 3D printing precision in generating anatomical models from patient-specific data has dual benefits including improved surgical training and preoperative planning. Realistic simulation of neurosurgical tasks can be developed using additive manufacturing processes such that skill acquisition is improved, and patient outcomes are positive. Innovative problem-solving using 3D printing received a wider range of responses, topping at 30%, rating it 2. This would suggest that while 3D printing is recognized as important, it is not yet integral to every participant’s training or practice, probably because of its recent introduction or unfamiliarity with the technology.

Research has often been central to the goals that decide career choices and build surgical skills in medical students. Awad et al. (2016) [11] note that this trend is notably reflected in the number of medical students who receive research grants, of which more than 50% go on to pursue a residency in neurosurgery. Additional support for this view is inferred from the fact that 40% of the participants rated the lab’s role in research skills as 4, and 30% rated it as 5. Likewise, leadership and decision-making skills promoted by the non-medical resources of the lab are also rated to be four by 50% of the participants and rated to be five by 20%. This shows that the role of the laboratory goes beyond technical skills to include research competencies regarding professional development. 60% of all the participants highly rated the lab’s contribution to fostering innovation. The entire neurosurgical residency is six years in length, during which time the critical emphasis in each year has been tailored, almost in a pillar-like fashion. Lab training is organized in such a way that it complements this pillar development and progressively increases in intensity from foundational-level lab skills to more advanced surgical-based decision-making as the years progress.

The integration of neurosurgery into the curricula of medical schools remains an important underdeveloped feature in the medical education system worldwide. Lee et al. (2020) [12] study indicated that the level of neurosurgical exposure varied grossly across different regions, with only 39.7% of students reporting any form of neurosurgical experience during their education. The idea is that regular use of the lab enhances participants’ ability to innovate in their practice. Kato et al. (2020) [1] review global disparities in neurosurgical education between developed and developing countries. Despite advances in surgical techniques or diagnostic tools, many developing regions still face huge barriers, including access to limited resources, training, and modern technology. They also advocate for international collaboration to close these gaps and support programs. Kanmounye et al. (2020) [13] discuss how the role of the Foundation for International Education in Neurological Surgery has transformed to decrease global neurosurgical disparity through education. Until recently, FIENS, founded in 1969, focused on brief mission trips but, since then, has transformed into a more sustainable model through the education of local neurosurgeons and the establishment of residency programs in LMICs. This model, labeled “service through education,” has enhanced the development of neurosurgical systems in LMICs and has led to a sustainable effect due to local ownership and international cooperation.

### Limitations

This study has several limitations. The sample size is relatively small, limiting the generalizability of findings to a broader population of neurosurgeons and trainees. Additionally, the study relies on self-reported data, which may introduce response bias. The cross-sectional design does not allow for the assessment of the long-term impacts of the neurosurgical lab on clinical outcomes. The survey instrument was developed specifically for this study and has not been previously validated, which may influence the reliability and interpretability of the results. Moreover, all participants were active users of the lab, which may introduce selection bias and lead to an overestimation of satisfaction and perceived benefit. Furthermore, while the study highlights the effectiveness of simulation-based training, it does not compare outcomes with traditional training methods. Future research should incorporate larger cohorts, objective skill assessments, and longitudinal follow-up to validate these findings.

## Conclusion

The neurosurgical virtual laboratory of the Neuroscience Hospital has become a means of developing skills, a platform for innovation, and nourishment of research and leadership for its users. Data from this study reveal that participants are satisfied and that the lab serves both routine training and higher levels of innovative skills. Such findings emphasize that Curtis’ latest technologies, such as 3D printing, should be continually updated and further integrated into neurosurgical training to better equip residents and surgeons for the specialty’s future challenges.

Future directions should focus on the development of validated assessment tools to objectively measure skill acquisition and clinical performance following simulation-based training. Incorporating multi-center studies, longitudinal tracking of residents’ progress, and comparisons with traditional training methods will be essential to further establish the effectiveness and scalability of this educational approach in neurosurgery.

## Electronic supplementary material

Below is the link to the electronic supplementary material.

Supplementary Material 1

## Data Availability

The datasets generated and analyzed during the current study are available from the corresponding author upon reasonable request.

## Abbreviations

3D: Three-Dimensional
χ^2^: Chi-Square Test
FIENS: Foundation for International Education in Neurological Surgery
LMICs: Low-and Middle-Income Countries
N: Sample Size
p: Probability Value
VR: Virtual Reality
