# Supplementary material for: Innovations in neurosurgical education: the role of neurosurgical labs at neuroscience hospital of Baghdad in enhancing surgical skills
Source: BMC Med Educ. 2025 Jul 6;25:1010. doi: 10.1186/s12909-025-07535-7 (PMC12232704; doi:10.1186/s12909-025-07535-7)
Supplement: Supplementary file 1 — Supplementary Material 1 [file 12909_2025_7535_MOESM1_ESM.docx]

**Assessment of Skills and Experience in the Neurosurgery Laboratory: A Survey for Neurosurgeons, residents and medical students**

**Section 1: Demographic Information**

1. **Age:**

- Under 25
- 25-30
- 31-35
- 36-40
- 41-45
- 46-50
- Over 50

1. **Gender:**

- Male
- Female

1. **Current Position:**

- Neurosurgical Resident
- Neurosurgeon
- Medical student
- Other (Please specify): _______

1. **Years of Experience in Neurosurgery:**

- 0-2 years
- 3-5 years
- 6-10 years
- 11-15 years
- More than 15 years

1. **Primary Role in the Lab:**

- Clinical Training (e.g., skill development, surgical simulations)
- Research (e.g., data collection, innovation)
- Education (e.g., teaching, mentoring)
- Administrative (e.g., lab management, coordination)
- Other (Please specify): _______

1. **Frequency of Lab Use:**

- Daily
- Weekly
- Monthly
- Occasionally
- Rarely

1. **What are your long-term career goals? (Select all that apply):**

- Clinical Practice
- Academic Medicine
- Research
- Administration/Leadership
- Industry (e.g., Medical Devices, Pharmaceuticals)
- Other (Please specify): _______

**Section 2: Lab Equipment and Resources**

1. **Rate the quality and functionality of the following lab equipment:**
   - **Microscope:**
     - 1 (Poor)
     - 2
     - 3
     - 4
     - 5 (Excellent)
   - **Training Models (e.g., anatomical models, simulators):**
     - 1 (Poor)
     - 2
     - 3
     - 4
     - 5 (Excellent)
   - **3D Printers:**
     - 1 (Poor)
     - 2
     - 3
     - 4
     - 5 (Excellent)
   - **Medical Books (e.g., neurosurgery textbooks, atlases):**
     - 1 (Poor)
     - 2
     - 3
     - 4
     - 5 (Excellent)
   - **Non-Medical Books (e.g., books on leadership, time management):**
     - 1 (Poor)
     - 2
     - 3
     - 4
     - 5 (Excellent)
2. **Specific Feedback on Equipment:**
   - Which equipment do you find most beneficial, and why?
   - Are there any pieces of equipment that you found inadequate or underutilized?

**Section 3: Training, Skill Development, and Educational Value**

1. **Assess the following specific skills gained through the use of lab resources (Rate each on a scale of 1 to 5):**
   - **Microsurgical Skills (e.g., suturing, dissection) using the Microscope:**
     - 1 (No Improvement)
     - 2
     - 3
     - 4
     - 5 (Significant Improvement)
   - **Anatomical Precision and Spatial Awareness using Training Models:**
     - 1 (No Improvement)
     - 2
     - 3
     - 4
     - 5 (Significant Improvement)
   - **Innovative Problem-Solving Skills through 3D Printing Technology:**
     - 1 (No Improvement)
     - 2
     - 3
     - 4
     - 5 (Significant Improvement)
   - **Diagnostic Accuracy (e.g., interpreting radiological images) using the Microscope and Models:**
     - 1 (No Improvement)
     - 2
     - 3
     - 4
     - 5 (Significant Improvement)
   - **Research Skills (e.g., data collection, analysis) supported by available resources:**
     - 1 (No Improvement)
     - 2
     - 3
     - 4
     - 5 (Significant Improvement)
   - **Leadership and Decision-Making Skills fostered by Non-Medical Books and Team Exercises:**
     - 1 (No Improvement)
     - 2
     - 3
     - 4
     - 5 (Significant Improvement)
2. **Educational Value:**
   - How well does the lab facilitate hands-on learning and the acquisition of practical skills?
     - 1 (Very Poorly)
     - 2
     - 3
     - 4
     - 5 (Very Well)
3. **What specific skill(s) have you developed most significantly as a result of using this lab?**

**Section 4: Research and Innovation**

1. **Have you utilized the 3D printers for developing new surgical techniques or models?**
   - Yes
   - No
2. **If yes, describe a specific instance where 3D printing directly contributed to your surgical practice or research.**
3. **Rate the lab's role in encouraging innovation and creativity in neurosurgical practices:**
   - 1 (Not at All)
   - 2
   - 3
   - 4
   - 5 (Extremely Encouraging)
4. **What additional resources or equipment would enhance the lab's capacity for innovation and research?**

**Section 5: Overall Experience and Suggestions**

1. **Overall Satisfaction with the Lab Experience:**
   - 1 (Very Dissatisfied)
   - 2
   - 3
   - 4
   - 5 (Very Satisfied)
2. **Main Strengths of the Neurosurgery Lab:**
   - What do you consider the most valuable aspects of this lab?
3. **Areas for Improvement:**
   - Where do you see the most need for enhancement?
4. **Recommendation:**
   - Would you recommend the use of this lab to other neurosurgeons or residents? Why or why not?
5. **Additional Comments or Suggestions:**
   - Please provide any further insights or recommendations for improving the neurosurgery lab.
